# Variety of Identifying and Assessing Preferences of Everyday Living of Older Adults

**DOI:** 10.1093/geroni/igab046.1014

**Published:** 2021-12-17

**Authors:** Martina Roes, Kimberly Van Haitsma

## Abstract

Identifying preference of older adults supports person-centred care. The most sophisticated instrument is the preference for everyday living inventory (PELI). The PELI has been translated into German language and tested in different care settings. For people who experience difficulties communicating their preference the PELI has been combined with photographs. The voice of older immigrants could lead to an enhancement of the PELI as well other preference tools. Thus, our symposium title: Variety of identifying and assessing preferences of everyday living of older adults. Our symposium includes four presentations: Dr. Bergmann will present data from a preference study in three different care settings (long-term care, nursing homes, adult day care) in Germany. The results indicate that the importance of certain preferences distinguishes between the care settings. Dr. Vanessa Burshnic will present data from her content validity study of photographs used to supplement the Preferences for Everyday Living Inventory-Nursing Home (PELI-NH) from the perspective of older adults. Content analysis revealed thematic codes describing participants’ photograph preferences including image quality, context, subject diversity, and relevance to long-term care. Mike Rommerskirch-Manietta will present results from a review to identify Instruments which can be used to assess preferences for everyday living of older adults. Interestingly instruments either represent multiple or only one domain. The study from Viktoria Peters-Nehrenheim does focus on preferences of older immigrants. She will present results how older immigrants (first generation) define preferences and how they can be assessed. Prof. Van Haitsma will be our discussant.

